# Current Status of Metabolomic Biomarker Discovery: Impact of Study Design and Demographic Characteristics

**DOI:** 10.3390/metabo10060224

**Published:** 2020-05-29

**Authors:** Vladimir Tolstikov, A. James Moser, Rangaprasad Sarangarajan, Niven R. Narain, Michael A. Kiebish

**Affiliations:** 1 BERG, Precision Medicine Division, Framingham, MA 01701, USA; Vladimir.Tolstikov@berghealth.com (V.T.); Rangaprasad.Sarangarajan@berghealth.com (R.S.); Niven.Narain@Berghealth.com (N.R.N.); 2 Beth Israel Deaconess Medical Center and Harvard Medical School, Boston, MA 02215, USA; ajmoser@bidmc.harvard.edu

**Keywords:** metabolomics, biomarker, demographics, clinical, phenotype, discovery

## Abstract

Widespread application of omic technologies is evolving our understanding of population health and holds promise in providing precise guidance for selection of therapeutic interventions based on patient biology. The opportunity to use hundreds of analytes for diagnostic assessment of human health compared to the current use of 10–20 analytes will provide greater accuracy in deconstructing the complexity of human biology in disease states. Conventional biochemical measurements like cholesterol, creatinine, and urea nitrogen are currently used to assess health status; however, metabolomics captures a comprehensive set of analytes characterizing the human phenotype and its complex metabolic processes in real-time. Unlike conventional clinical analytes, metabolomic profiles are dramatically influenced by demographic and environmental factors that affect the range of normal values and increase the risk of false biomarker discovery. This review addresses the challenges and opportunities created by the evolving field of clinical metabolomics and highlights features of study design and bioinformatics necessary to maximize the utility of metabolomics data across demographic groups.

## 1. Introduction

Metabolomics is an emerging field of “omics” that characterizes small molecule metabolites in biological systems (http://metabolomicssociety.org/). Metabolomics analyses reflect both the steady-state physiological equilibrium of cells or organisms as well as their dynamic metabolic responses to environmental stimuli [1,2,3,4,5]. In comparison to other human biological measurements, the metabolome is uniquely suited to depict the phenotype and measure the impact of environmental factors on the end products of metabolism [2]. The field has expanded rapidly since its inception more than two decades ago as a result of parallel improvements in bioanalytical platforms and methods for data analysis [1,2,3,4,5].

**Figure 1:**
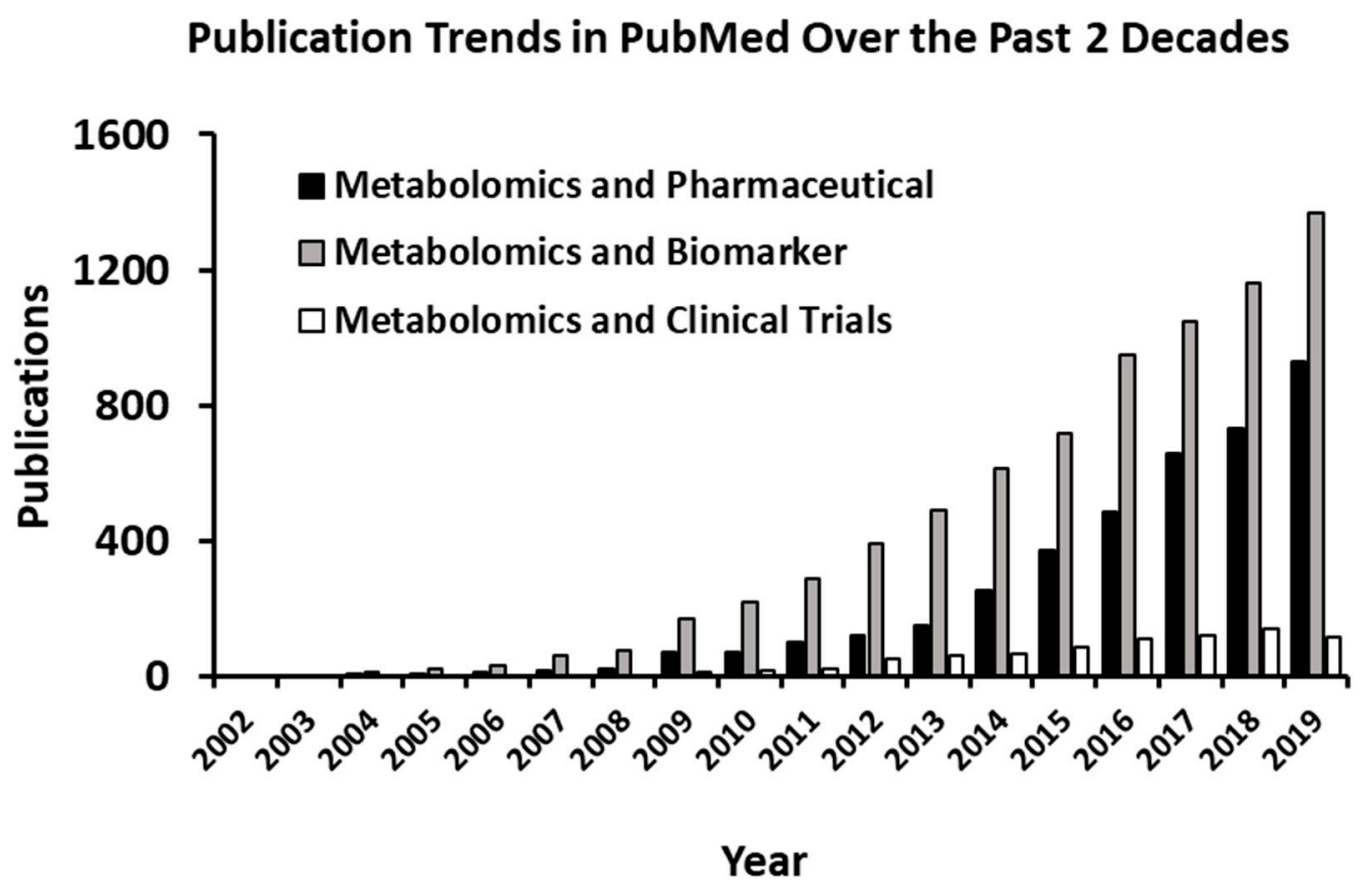
Results of PubMed publications surveillance of biomarkers, pharmaceutical development, and clinical trials.

Current methods of clinical decision support rely on a limited selection of diagnostic biomarkers to monitor response to treatment in individual patients. Human metabolites serve as valid clinical markers and are listed in the Mayo Clinic test catalog at https://www.mayocliniclabs.com/test-catalog/, LabCorp test menu https://www.labcorp.com/test-menu/search, and Quest Diagnostics Test Directory https://testdirectory.questdiagnostics.com/test/home [4]. Many of these biomarkers were identified decades ago before metabolomics was invented. By comparison, the Human Metabolite Database (HMDB, https://hmdb.ca/) now catalogs more than 25,000 metabolites in human blood [1]. Publications referencing metabolomics assisted biomarker discovery demonstrate exponential growth in utilization (Figure 1) by academia and industry. A search for the word metabolomics in U.S. National Library of Medicine at Clinical Trials retrieved 962 studies (https://clinicaltrials.gov/) and an almost equal number of completed or recruiting clinical studies.

The human metabolome is composed of endogenous and exogenous compounds and their respective metabolites, which originate internal and external to the host. Food components, microbiome products, supplements, and pharmaceuticals are examples of sources of human exogenous metabolites. Recent evidence links the microbiome to alterations in the human metabolome that impact human health [6,7,8]. For example, gut microorganisms can induce excess production of uremic toxins [9], like indoxyl sulphate, p-cresol sulphate, and trimethylamine-N-oxide, which accumulate in the circulation as markers of renal dysfunction [8]. Multi-omic profiling of the fecal microbiome demonstrates a strong association with visceral fat mass and abdominal obesity only modestly influenced by the host. A similar relationship between the gut microbiome, diet, and the metabolome has been observed. In particular, plant-derived nutrients are associated with alterations in circulating metabolites such as bile acids [7], indicating potential relationships between the composition of the gut microbiome and resulting dietary impacts on host metabolism.

Technological advancements have enabled novel strategies to discover individual biomarkers as well as panels of markers derived from different -omics platforms [5,6,7,8,9,10,11,12,13,14]. These technologies harvest substantial volumes of data at multiple biological levels and sift the resulting large data sets with informatics tools including artificial intelligence (AI). This intriguing approach has accelerated biomarker discovery and routinely incorporated in preclinical study workflows, including for the search for potential novel therapeutic targets and candidate drugs [15,16,17,18,19,20,21,22,23,24,25,26,27,28,29,30,31,32,33]. The capability of metabolites to illustrate varied biological processes, such as cell differentiation and maturation, insulin signaling, T-cell survival, and macrophage immune responses is reported and extensively reviewed [20,21]. Commercial services to identify relevant metabolic biomarkers, investigate biochemical pathways, and report the metabolic state of a cell, tissue, or living organism, have become mainstream (https://www.biocrates.com/services/metabolomics-services). The data allow research institutions and commercial organizations to identify metabolites with active and passive effects on phenotypes of interest. The field of clinical metabolomics will continue to evolve as GCLP standards for CLIA laboratories remain under development. With sufficient scientific rigor and validation, metabolomic profiles may acquire FDA approval for clinical use and monitoring of therapy.

## 2. Results

### 2.1. Current Challenges 

Major challenges exist translating metabolomic biomarkers from discovery to application in population health studies that characterize human biology focused on systemic metabolism [2,3,4,32,33,34,35]. Wide chemical diversity and the presence of isomers and isobaric compounds prevent a single bioanalytical platform from providing all-inclusive discovery of metabolites in a biological system. Therefore, a combination of orthogonal separation techniques, including gas and liquid chromatography coupled with mass spectrometry, must be employed in global metabolite profiling protocols. Separation of hydrophobic analytes requires application of reverse phase and hydrophilic chromatography to separate polar metabolites. Increased metabolome coverage may be achieved with the different ionization techniques such as electrospray, chemical, and electron impact. Detected metabolites must be identified through chemical standard analysis and characterized by method-specific retention times as well as mass spectrometry measurements like monoisotopic mass and fragmentation patterns. The absence of authenticated standards requires that detected features be annotated by matching to known parameters and mass fragmentation patterns within existing mass spectrometry databases like (METLIN (http://metlin.scripps.edu/index.php), HMDB (http://www.hmdb.ca/), MASSBANK (http://www.massbank.jp/Index), NIST-MS (http://chemdata.nist.gov/), IDEOME (http://mzmatch.sourceforge.net/ideom.php), and mzCloud (https://mzcloud.org/).

The field has evolved rapidly due to the increasing use of targeted metabolomics. Targeted approaches provide reliable identification and simultaneous quantification of hundreds of metabolites made possible by developments in tandem mass spectrometry and high-resolution high mass accuracy mass spectrometry [2,3,4,5]. Technology notwithstanding, extensive quality control and quality assurance protocols are mandatory. Standard operation procedures (SOP) must be implemented to reduce the preanalytical variation and batch to batch variability of data in a metabolomics workflow. Preanalytical variation is rooted in sample harvesting, storage, and transportation. Therefore, it is highly desirable to implement testing at biobanks to reduce the risk of study failure and allow early identification of metabolites that demonstrate excessive preanalytical variation. For example, there is no accepted procedure to limit the impact of hemolysis on plasma, serum, and buffy coat samples.

Significant degradation of metabolites can occur if existing SOPs are not followed, such as quenching protocols to inhibit the degradation of metabolites during tissue harvest and extraction, particularly in relation to in vitro studies [2,3,4,5]. Although biofluid harvest and storage protocols are standardized in most clinics and hospitals, the processes may vary between countries and different regions. The need to achieve standardization in metabolomics resulted in the formation of the metabolomics quality assurance and quality control consortium (mQACC; https://epi.grants.cancer.gov/Consortia/mQACC/). mQACC was established in February 2018 to address key quality assurance (QA) and quality control (QC) issues in the untargeted metabolomics field among relevant stakeholders in academia, industry, and government institutions.

### 2.2. Assessment of Sample Size Requirements for Clinical and Translational Research

A recent large-scale study in clinical metabolomics demonstrated the importance of insufficient sample size on the reproducibility of results [23]. Mass spectrometry-based metabolomics was used to establish baseline levels and variation in the sera of 1200 adults from the United Kingdom. The authors evaluated three groups based on age, sex, and BMI to test their hypothesis “that many biological studies are underpowered with regard to their ability to come to a robust and statistically significant and justifiable biological conclusion” [23]. Not unexpectedly, median accuracy increased, and variation decreased, as sample size approached 600, a sample size that was representative of the whole sample population.

Subsequent data estimated statistical power and sample size in metabolic phenotyping and demonstrated complex relationships between sample size, power, and effect size for real multivariate data sets [24]. Using univariate and multivariate approaches, the authors projected a power of 0.8 at a sample size of 200, whereas a sample size of 300 was required to obtain 0.95 power. The authors observed that attributed variations in the results of prior biological studies to datasets using a low minimum sample size of around 20 samples. The authors extended their approach to both nuclear magnetic resonance and liquid chromatography−mass spectrometry data from human cohorts (1861 urine samples (ARIC study) and 951 plasma samples (AIRWAVE study)). A recent review in Drug Discovery Today suggested that a power of 0.8 become the benchmark for effective biomarker discovery in precision medicine [14]. Employing the power analysis module in MetaboAnalyst 4.0 (https://www.metaboanalyst.ca), 63 urine metabolites were measured among 77 samples in two groups of cancer patients. In order to achieve the desired power of 0.84 at a false discovery rate (FDR) 0.1, the estimated sample size was 400 per group.

Despite these strong recommendations concerning sample size, peer-review journals report pilot clinical -omics studies with insufficient sample size. For instance, a recent study of the mechanism by which APOA2-saturated fat intake affects obesity [25] was validated across several populations in an epigenome-wide association study. The authors assumed that a sample size of 20 for each CC and TT genotype was sufficient to provide 95% statistical power to detect epigenetic signatures. This assumption proved false for the metabolomics analysis due to the well-known impact of environmental factors on human metabolomics studies. Despite *p*-value calculations using ANCOVA contrast in a two-way ANOVA model adjusting BMI for metabolite levels, FDR adjustment was not performed during the metabolomics data analysis. Longitudinal datasets may augment underpowered studies by demonstrating dynamic changes over time that are associated with actionable changes. Bayesian analytical approaches to causal inference may increase the effectiveness of biomarker identification by comparison to standard statistical association.

### 2.3. Demographic Impacts

Although the effects of sample size and sample quality can be minimized by adopting strict protocols, demographic variability must be addressed independently during metabolite biomarker discovery. Dunn et al. [23] set the benchmark for evaluating the effect of demographics and sample size on phenotyping quality. This study conducted as a part of The Husermet project utilized mass spectrometry to analyze the metabolome of human serum. Demographic factors had an important impact on metabolic changes associated with sex, age, BMI, blood pressure, and smoking habits of the participants. A significant number of metabolites were associated with insulin resistance, and metabolic syndrome increased with age. Correlations between metabolite profiling and clinical chemistry were discovered, such as circulating HDL cholesterol and levels of fatty acids, di- and triglycerides, liver function, uric acid levels, etc. A recent cross-sectional study measured 106 metabolites in human plasma from 2503 healthy subjects [34]. Metabolites from multiple classes, including amino acids, lipids and acylcarnitines were monitored because of their involvement in core metabolic processes. Interestingly, liver function had the strongest association with plasma metabolite profiles, followed by kidney function and insulin resistance. In this study, the authors established the main clinical covariates associated with differences in the plasma metabolite profiles. Authors used principal component analysis (PCA) to reduce the large number of metabolites into independent components. Results suggest that liver function demonstrates the strongest association with the major differences in the plasma metabolite profiles measured in this study in particular lipid profiles, including cholesterol. Medium-chain acyl carnitines mainly contributed to components associated with hypertension and kidney function. Associations between the short list of measured metabolites and specific patient phenotypes suggest that multiple independent metabolic mechanisms may contribute to recognized clinical conditions. One possible limitation on the external validity of this study was the homogeneous population of patients from a specific region in Sweden. A recent Australian study acquired blood samples from 1180 children and 1325 parents and analyzed amino acid species, lipoprotein subclass measures, lipids, and fatty acids as composite biomarkers of inflammation and energy homeostasis [35]. Applied in this study NMR profiling technique demonstrated metabolic measures for each participant that segregated the population according to age and sex.

Longitudinal metabolomic studies have confirmed impacts of age and sex on metabolite profiles. A total of 2344 plasma samples in fasting patients from the longitudinal Wisconsin Registry for Alzheimer’s Prevention (WRAP) study were analyzed from 1212 participants initially free of Alzheimer’s disease at enrollment. After corrections for multiple testing, 56.8% of measured metabolites were associated with age and 63.4% with sex. Genome-wide screening to evaluate the role of hereditary factors on metabolite profiles demonstrated significant influence by a complex combination of genomic and environmental factors [36,37,38,39]. A cross-sectional study evaluating associations between the human lipidome and age enrolled 100 subjects with an apolipoprotein E (APOE) E3/E3 genotype aged 56–100 years. After rigorous statistical analysis, a strong negative association between age and lipid levels was observed that predominated in male compared to female subjects [36]. Sex and age-related differences were also found in a study of elderly subjects with pre-frailty sub-phenotypes [38]. Further, metabolomic analysis has been used to investigate gait speed in the Baltimore Longitudinal Study of Aging (BLSA). Baseline and follow-up measurements were from 504 adults aged 50 or older over a median follow-up of 50.5 months. Plasma metabolites were measured by targeted mass spectrometry using a commercially available metabolomics kit (Absolute IDQ p180 Kit, Biocrates). Low plasma levels of LPC 18:2 was an independent predictor of decline in gait speed in older adults [38]. LPC 18:2 is associated with impaired glucose tolerance, insulin resistance, type 2 diabetes, coronary artery disease, and memory impairment. These meticulous studies (Table 1) make the critical link between demographic characteristics and phenotyping by omics protocols.

Although animal models permit strict control over the study population and environmental conditions, demographic variation persists. Despite controlled genetics, age, sex, feeding, and environmental conditions among animals in the same cohort, variation in metabolite levels is commonly observed and must be addressed through increased sample size as in human studies. One design consideration to overcome these limitations of metabolomics is to recruit demographically balanced cohorts, a strategy of importance to longitudinal studies of difficult diseases like Alzheimer’s (AD). If demographically balanced cohorts are not possible, exclusion of metabolite markers for biomarker assessment that correlate with demographics should be removed for further development. Serum-based liver function markers and cerebrospinal fluid (CSF) biomarkers were measured during 8 consecutive years in a longitudinal study of 1581 AD participants. Associations of liver function markers with AD were assessed using generalized linear models adjusted for confounding variables and multiple comparisons. Although liver function had the most intriguing association with AD [40], liver function also was most strongly associated with the plasma metabolite profile among healthy subjects [34]. Integrating analyses of genomics, longitudinal metabolomics, and Alzheimer’s risk factors in 1111 cohort participants revealed that changes in CSF metabolite levels explained more than 60% of variance in CSF levels of tau, a detrimental protein that accumulates in the brain of AD patients and is necessary for diagnosis [40].

### 2.4. Metabolic Markers in Clinical Studies

The metabolome is directly linked to perturbations in cell metabolism caused by environmental stimuli as well as disease, thus metabolomics has been successfully used to measure phenotypic outcomes in vitro and in vivo [15,16,17,18,19,20,21,22,23,24,25,26,27,28,29,30,31,32,33,40,41,42,43,44,45,46,47,48,49]. Examples include observations of oncometabolites, such as 2-hyroxybutyrate, sarcosine, choline, succinate, lactate, fumarate, and glucose, associated with tumor growth and metastasis that may also function as biomarkers for leukemia, renal carcinoma, breast, brain, or prostate cancer [26,27,28,29,30,31,32]. Metabolomics may also be used to accumulate additional insights during studies of clinical pharmacology and biomarker discovery [3,4,14,15,16], recognizing that large scale and multi-cohort studies will increase the discovery of candidate biomarkers.

Advances in bioanalytical capability has expanded the number of metabolites discovered in retrospective and prospective studies that form the basis for existing clinical diagnostic assays. A recent report validated a rapid, repeatable, and precise method for extracting and quantifying 32 eicosanoid urinary metabolites by LC–MS/MS [42]. These signaling mediators were rapidly metabolized and excreted as a mixture of primary and secondary metabolites, a finding that has expanded interest in human urine as a useful, readily accessible biofluid for monitoring the endogenous synthesis of these molecules despite the inherent difficulties that urine’s chemical complexity poses for normalizing metabolite results. Human urine contains a candidate panel of 17 metabolites potentially useful as a biomarker of colorectal cancer (CRC). The performance of the panel was tested among 342 participants (CRC, 171; healthy controls, 171) in Canada and the United States) by comparison to standard of care colonoscopy and histopathology. Targeted liquid chromatography–mass spectrometry was performed to quantify urine metabolites. Multiple statistical models demonstrated sufficient separation of CRC from controls, opening up an excellent opportunity for translation to clinical diagnosis [43].

In another example, targeted clinical metabolite profiling was used to stratify patients with diabetes [43]. An LC–MS/MS platform quantified and discovered new, clinically relevant metabolites. One such marker is Trimethylamine N-oxide (TMAO), a newly identified gut microbiota-dependent metabolite that contributes to a variety of conditions, such as diabetes, atherosclerosis, and cardiovascular disease. TMAO is also a prognostic marker of unfavorable clinical outcomes among patients with acute ischemic stroke [45,46].

Modern metabolomics studies have considerably benefited from improvements in study design that minimize the uncontrolled effects of demographic variation and sample size and account for unique sources of bias. These lessons apply to both observational and interventional studies as well as those with multi-arm or multi-stage designs. Study recruitment must balance demographic characteristics among participants in each cohort and provide sufficient sample size to satisfy requirements for sufficient statistical power at the anticipated false discovery rate. For example, a cohort of 50 type 1 diabetics underwent measurements of amino acids like tyrosine and homocitrulline previously linked to participant demographic characteristics [44]. No age or BMI dependent changes in tyrosine, tryptophan, glutamine, or phenylalanine levels were reported, contradicting prior studies conducted with a larger sample size (Table 1).

Given the recognized link between population demographics and biomarker discovery, the biotechnology industry has used complex statistical methodologies to control for the confounding influences of sex, age, and BMI on metabolomics data generated from study participants and healthy controls. A prominent example is the US patent issued to Metanomics GMBH (Germany) in 2013 in its search for a quantitative assay kit for metabolite biomarkers for pancreatic cancer [47]. This patent protected three critical aspects related to precision medicine for pancreatic cancer: a novel method for diagnosis, treatment selection, and evaluation of treatment outcome. The patent described numerous small molecule biomarkers as candidates to differentiate pancreatic carcinoma from liver cirrhosis and pancreatitis. Statistical analysis was performed using a simple linear model (ANOVA) with the following fixed effects: Disease, body mass index, age, storage time, and recruitment site. A long list of candidate markers was supported by statistical evidence for their significance and included coenzymes, carboxylic acids, fatty acids, amino acids, saccharides, sugar alcohols, and lipids Metanomics GMBH (Germany) published a metabolic signature of nine molecules discriminating pancreatic ductal adenocarcinoma from chronic pancreatitis in 2017 [48]. The signature was selected based on the multivariate elastic net analysis comprising of the biomarker panel, and their univariate statistical analysis performed by a linear model on log base 10-transformed data considering disease status, sex, body mass index, age, and storage time as fixed effects. Weaknesses of their approach included limited sample size and failure to balance sex between the cohorts, which may compromise the clinical utility of this marker panel in commercial use. The proposed panel listed histidine as a candidate biomarker despite its known interactions with age and body mass index (Table 1), as well as phosphatidylcholine and sphingomyelins levels that correlate with sex and BMI (Table 1). Given the tiny fraction of total body mass represented by the underlying pancreatic cancer, it remains unclear whether metabolomics approaches by themselves are sufficiently specific as a diagnostic and prognostic tool in the absence of other complementary -omics approaches, which account for the confounding effects of metabolic processes in the normal major organs.

The Bogalusa Heart Study is an example of a successful demographically balanced study to find cognition phenotypes among its participants [49]. Metabolite profiling of plasma samples from 1177 participants was performed by Metabolon, Inc. (USA) and linked to cognition. Multiple linear regression was used and adjusted for demographics. Weighted correlation network analysis evaluated correlations between cognition and modules of co-abundant metabolites after adjustment for covariables, including age, sex, ethnicity, cigarette smoking, drinking, education, depression, vocabulary, BMI, systolic blood pressure (SBP), LDL-C, and glucose. In contrast, several small metabolomics studies claim to have discovered a wide variety of metabolic biomarker candidates, which have not been successfully validated nor approved by the FDA (FDA official site, 16 April 2019) [14].

One technical approach to improve trust in metabolomics is the introduction of replication studies [50,51,52]. Such studies are demonstrating demographical balance in both the discovery and validation cohorts and may account for different geographical regions. Statistical power is considered in parallel. An example of this approach is a mass spectrometry-based metabolite profiling study to identify potential markers of colorectal cancer detection in circulation [50]. The Geijsen study used a discovery replication design to screen two independent, relatively large, patient cohorts of Caucasian origin from two different countries. Plasma samples from 268 colorectal cancer patients and 353 controls were analyzed using separate discovery and replication sets from two European cohorts (ColoCare Study: *n* = 180 patients/*n* = 153 controls; the Colorectal Cancer Study of Austria (CORSA) *n* = 88 patients/*n* = 200 controls). Multiple logistic regression was used to test the association between disease state and metabolites. Potential markers were validated in the replication set and adjusted for sex, age, BMI, and smoking status to assure robustness of the resulting panel, which identified changes in the levels of several amino acids and lipids. Interestingly, changes in valine were inversely proportional to age and BMI, suggesting that age and BMI could not account for decreased circulating levels of valine among CRC patients (Table 1). Conversely, levels of leucine and LPCs were associated with age and BMI as shown in Table 1. The results may illustrate early metabolic changes during colorectal carcinogenesis rather than the process of metastatic spread and do not provide insight into causality. Another similar study investigated circulating markers for incident Type 2 Diabetes between two Swedish prospective cohorts comprising > 4000 study participants. Independent, replicable associations were discovered between the future risk of T2DM and three metabolites: N2,N2-dimethylguanosine, 7-methylguanine, and 3-hydroxytrimethyllysine [51]. Another Swedish study used plasma or serum samples from three community-based cohorts to identify metabolites associated with incident heart failure in a general population (total *n* = 3924; 341 incident heart failure events; median follow-up ranging from 4.6 to 13.9 years) and discovered associations between heart failure and circulating levels of urobilin and sphingomyelin (30:1) [52]. Elevated levels of sphingomyelin (30:1) linked to heart failure could not be explained by known associations between BMI and sphingomyelin (SM) in other studies (Table 1). Thus, this process exemplifies the optimal strategy to improve translational utility of the data and validation of the findings.

## 3. Conclusions/Recommendations

Despite its inherent complexities and challenges, metabolomics is emerging as an important tool to characterize patient phenotypes in parallel with other -omics platforms [12]. The establishment and incorporation of optimized clinical study design, standardized protocols and quality metrics, and utilization of independent demographic factors will allow for efficient translation of actionable metabolite biomarkers impacting clinical trials and population health studies. Further, power calculations should be performed to assess confidence in the biomarker discovery output. As referenced in Dunn et al., which performed metabolomic assessment on samples from the UK biobank and determined utility of metabolites in stratifying demographic features powered at 0.8 for 200 samples and 0.95 for 300 samples. This study established the ideal reference point for discovery of biomarkers using known biological influence from demographics, which could be applied to powering studies evaluating markers for heterogeneous diseases. In this line, groups should further be balanced demographically, or a plan should be in place to exclude metabolite variables that correlate with demographic features. Additionally, cross validation in studies using multi-site cohorts further exemplifies the potential translational utility and should be encouraged in all studies. These advances and strategies in metabolomic biomarker discovery promise to improve multidimensional assessment of a population’s health while accelerating drug discovery and the adoption of precision medicine into general clinical practice.

## Figures and Tables

**Table 1:** Major classes of metabolites affected by sex, age, and BMI.

Metabolite Class	Metabolite	Gender/F	Age	BMI	Clinical Relevance (Mayo Clinic)	Reference
Carboxylic acids	Citrate	☑ ⇧	☑ ⇧	☑ ⇩	Metabolic diseases ⇩	[23,36]
	Aconitate		☑ ⇧			[36]
	Urate	☑ ⇧			Acute uric acid nephropathy ⇧	[23,36]
	Hexadecenoic acid	☑ ⇧			Nutrients deficiency ⇩	[23,36]
	4-hydroxyphenyllactic acid	☑ ⇩				[23]
	Octadecadienoic acid		☑ ⇧		Nutrients deficiency ⇩	[23]
	Dodecanoic acid			☑ ⇩		[23]
Acylcarnitines	Butyrylcarnitine	☑ ⇩			Fatty acid beta-oxidation disorders ⇧	[37]
	Oleoylcarnoiitine	☑ ⇩	☑ ⇧		Fatty acid beta-oxidation disorders ⇧	[36]
	Palmitoylcarnitine	☑ ⇩	☑ ⇧		Fatty acid beta-oxidation disorders ⇧	[36]
	Eicosenoylcarnitine	☑ ⇩	☑ ⇧		fatty acid beta-oxidation disorders ⇧	[36]
Amino acids	Tyrosine	☑ ⇩	☑ ⇧	☑ ⇧	Inborn errors of metabolism ⇧	[23,36]
	Creatinine	☑ ⇧	☑ ⇧	☑ ⇧	Kidney disease/failure ⇧	[23,33]
	Methionine sulfoxide	☑ ⇧				[23]
	Serine	☑ ⇧	☑ ⇩	☑ ⇩	Inborn errors of metabolism ⇧	[23,36]
	Aspartate	☑ ⇩	☑ ⇩		Inborn errors of metabolism ⇧	[23]
	Tryptophan	☑ ⇩	☑ ⇩		Inborn errors of metabolism ⇧	[23,36]
	Methionine	☑ ⇩	☑ ⇩		Inborn errors of metabolism ⇧	[23]
	Threonine		☑ ⇩	☑ ⇩	Inborn errors of metabolism ⇧	[23,36]
	Cysteine		☑ ⇧	☑ ⇧		[23]
	Cystine			☑ ⇧	Inborn errors of metabolism ⇧	[23]
	Glutamine		☑ ⇧	☑ ⇧	Inborn errors of metabolism ⇧	[23,36]
	Phenylalanine	☑ ⇩	☑ ⇧	☑ ⇧	Inborn errors of metabolism ⇧	[23]
	Valine	☑ ⇩		☑ ⇧	Inborn errors of metabolism ⇧	[23,36]
	Leucine	☑ ⇩	☑ ⇩			[36]
	Histidine		☑ ⇩	☑ ⇩	Inborn errors of metabolism ⇧	[23,36]
	Phosphoserine		☑ ⇩	☑ ⇩		[23]
	2-aminomalonic acid			☑ ⇩		[23]
	Aminooctanoic acid	☑ ⇧				[23,38]
Lipids	DAG	☑ ⇧		☑ ⇩		[23]
	PC	☑ ⇧				[23]
	Glycerol	☑ ⇧	☑ ⇧	☑ ⇧		[23]
	Glycerol-3-phosphate	☑ ⇧	☑ ⇧	☑ ⇧		[23]
	Threitol		☑ ⇧			[23]
	Phosphate		☑ ⇧			[23]
	LPC			☑ ⇩		[23]
	SM			☑ ⇩		[23]
	Cholesterol		☑ ⇧	☑ ⇧		[33]
	TAG		☑ ⇧	☑ ⇧	lipoprotein metabolism ⇧	[33]
	LPE	☑ ⇩				[38]
Sterol lipids	Androgenic	☑ ⇩	☑ ⇩			[36]
Sugars	Mannose	☑ ⇩				[38]
	Fructose	☑ ⇩			inborn errors of metabolism ⇧	[38]
Nucleotides	N1-methylinosine	☑ ⇩	☑ ⇧			[36]
	5-methylthioadenosine	☑ ⇩	☑ ⇧			[36]
	Pseudouridine	☑ ⇩	☑ ⇧			[36]
Vitamins	Vitamin D		☑ ⇩		chronic renal failure ⇩	[23]

Major classes of metabolites affected by sex, age, and BMI. DAG—diacylglycerol, PC—phosphatidylcholine, LPC—lysophosphatidylcholine, SM—sphingomyelin, TAG—triacylglycerol, and LPE—lysophosphatidylethanolamine. In the sex columns, ⇧ means the metabolite level was higher in women (F), whereas ⇩ means it decreased. The corresponding level in men has the opposite trend. For all other columns, ☑ means reported dependence with the reference provided, and ⇧ means the metabolite level increased with age, or BMI, whereas ⇩ means it decreased with age or BMI. Clinical relevance column shows the assay for diagnosing disease involved with measurements of the corresponding metabolites with the expected direction of metabolite level changes in bio-fluids. ⇧ means the metabolite level increased with the onset of disease, whereas ⇩ means it decreased. For greater details we recommend exploration of the original articles.
